# Catenarin Prevents Type 1 Diabetes in Nonobese Diabetic Mice via Inhibition of Leukocyte Migration Involving the MEK6/p38 and MEK7/JNK Pathways

**DOI:** 10.1155/2012/982396

**Published:** 2012-02-13

**Authors:** Ming-Yi Shen, Yu-Ping Lin, Bei-Chang Yang, Yu-Song Jang, Chih-Kang Chiang, Clément Mettling, Zeng-Weng Chen, Joen-Rong Sheu, Cicero L. Chang, Yea-Lih Lin, Wen-Chin Yang

**Affiliations:** ^1^Agricultural Biotechnology Research Center, Academia Sinica, 128 Academia Road, Section 2, Nankang, Taipei, Taiwan; ^2^Institute of Basic Medical Sciences and Department of Microbiology and Immunology, National Cheng Kung University, Tainan, Taiwan; ^3^Department of Aquaculture, National Taiwan Ocean University, Keelung 20224, Taiwan; ^4^Department of Chinese Medicine, Buddhist Tzu Chi General Hospital, Hualien 970, Taiwan; ^5^Institut de Génétique Humaine (CNRS UPR-1142), 141 rue de la Cardonille, 34396 Montpellier Cedex 05, France; ^6^Department of Pharmacology, Taipei Medical University, Taipei 110, Taiwan; ^7^Department of Veterinary Medicine, National Chung Hsing University, Taichung 402, Taiwan

## Abstract

Inflammation contributes to leukocyte migration, termed insulitis, and **β**-cell loss in type 1 diabetes (T1D). Naturally occurring anthraquinones are claimed as anti-inflammatory compounds; however, their actions are not clear. This study aimed to investigate the effect and mechanism of catenarin on the inflammatory disease, T1D. Catenarin and/or its anthraquinone analogs dose-dependently suppressed C-X-C chemokine receptor type 4 (CXCR4)- and C-C chemokine receptor type 5 (CCR5)-implicated chemotaxis in leukocytes. Catenarin, the most potent anthraquinone tested in the study, prevented T1D in nonobese diabetic mice. Mechanistic study showed that catenarin did not act on the expression of CCR5 and CXCR4. On the contrary, catenarin inhibited CCR5- and CXCR4-mediated chemotaxis via the reduction of the phosphorylation of mitogen-activated protein kinases (p38 and JNK) and their upstream kinases (MKK6 and MKK7), and calcium mobilization. Overall, the data demonstrate the preventive effect and molecular mechanism of action of catenarin on T1D, suggesting its novel use as a prophylactic agent in T1D.

## 1. Introduction

T1D is a life-threatening disease arising from the destruction of insulin-producing *β* cells by leukocytes with a strong inflammatory reaction. Five to 30% of diabetic patients are diagnosed with T1D. Patients with T1D share many similar genetic and immunopathological features with nonobese diabetic (NOD) mice, an established mouse model for T1D study [1, 2]. During T1D development, leukocytes begin to invade the pancreatic islets of NOD mice [3]. T lymphocytes are main players in T1D though other leukocytes such as B lymphocytes, dendritic cells, macrophages, and NK cells are also implicated [4, 5]. Leukocyte infiltration into the pancreatic islets, called insulitis, contributes to a gradual loss of pancreatic *β* cells, leading to insulin insufficiency and deficiency, a hallmark of T1D [6]. This infiltration is thought to be regulated by chemokine receptors and chemokines [7–10].

Inflammation involves activation and directed migration of leukocytes from the venous system to the inflamed sites where chemokines are produced and released. Nonresolved inflammation is associated with a broad category of diseases, that is, autoimmune diseases, cancers, and so forth [11]. In recent years, molecular studies have identified a variety of proteins and chemical mediators implicated in inflammation. Among them, chemokines and their cognate receptors play an essential role in inflammatory response and therefore, could be potential drug targets for inflammatory diseases [12–15]. T cells are thought to be important players in T1D [16], although other leukocytes are also involved in the disease [4]. CXCR4 is expressed in naïve T cells and the other leukocytes [17]. CCR5 is preferentially expressed in activated T cells and macrophages [18–20]. Therefore, interference with CXCR4 and CCR5 could be a promising approach for insulitis and T1D prophylaxis and therapy. Chemokine receptors belong to a family of 7-helix transmembrane G-protein-coupled receptors (GPCRs). On chemokine engagement, chemokine receptors initiate the binding of G*α* subunit to guanosine triphosphate and the dissociation of G*α* subunit from G*βγ* subunit. This activates protein tyrosine kinases, mitogen-activated protein kinases (MAPKs), and phospholipase C. Secondary messengers, inositol triphospahte and diacylglycerol, which are converted from phosphatidylinositol by phospholipase C, increase calcium mobilization and activation of protein kinase C, respectively. The above biochemical event results in cell chemotaxis and other cell functions [21].

Inflammation is involved in insulitis and *β* cell destruction in T1D [6]. Thus, a couple of therapeutic agents such as cyclooxygenase-2 (Cox-2) inhibitors, acetylsalicylic acid and tenidap and immune modulators, FK506, lisofylline and cytopiloyne, were reported to prevent T1D by inhibiting a variety of inflammatory pathways in immune cells [22–25]. However, their antidiabetic efficacy has been unsatisfactory. Therefore, search for novel anti-inflammatory agent for T1D therapy is practically significant. Medicinal plants, used in complementary and alternative medicine worldwide, are a rich source of anti-inflammatory compounds [26]. Moreover, a recent review on traditional Chinese medicines suggests that Chinese herbal formulations with hypoglycemic and anti-inflammatory activities are useful to inhibit diabetes development [27]. Therefore, anti-inflammatory complementary and alternative medicine may be beneficial for T1D. Catenarin, cascarin, emodin, and rhein represent a class of naturally occurring anthraquinone compounds from medicinal herbs. Most of them are best known as active compounds found in laxative herbs, commonly used to treat constipation. Apart from the laxative activity, emodin, the most studied anthraquinone in the literature, was claimed to possess anti-inflammatory activity as well as anticancer, antimicrobial, diuretic, vasorelaxing and phytoestrogen activities [3, 28–31]. Emodin was shown to inhibit hepatitis [30], pancreatitis [32], and NF-*κ*B activation [33] in cells and animals, suggesting its anti-inflammatory property. Another anthraquinone, rhein, is an active metabolite of diacerhein, a commercial drug used for osteoarthritis [34]. These publications suggest that anthraquinone compounds may have anti-inflammatory activity. Nevertheless, the anti-inflammatory effect and mechanism of the anthraquinones on T1D and other inflammatory diseases are poorly understood.

In this study, we examined the effect of catenarin and its derivatives on CCR5- and CXCR4-implicated chemotaxis in T cells. We then evaluated the *in vivo *effect of catenarin on leukocyte migration during T1D in NOD mice. Finally, we investigated the likely mechanism by which catenarin inhibited chemotaxis in T cells.

## 2. Materials and Methods

### 2.1. Drug Administration, Diabetes Measurement, Immunohistochemistry, and Cellularity Analysis

NOD mice were maintained according to the institutional animal care and utilization committee (OMiIBAYW20100043). Female NOD mice were intraperitoneally injected with a dose of catenarin (4, 20 and 40 mg/kg body weight (BW)), acetylsalicylic acid (40 mg/kg), or vehicle (0.1% dimethyl sulfoxide (DMSO), negative control) three times per week, from 4 to 30 weeks of age. As previously published [24], glycosuria and glycemia in the above mice were monitored every week using a Clinistix strip (Bayer, Pittsburgh, PA, USA) and an Elite glucometer (Bayer), respectively. Animals are considered diabetic when blood glucose levels were >250 mg/dL for 2 consecutive weeks. The percentage of glycosylated hemoglobulin A1C (Hb_A1C_) in blood samples was measured using a DCA 2000 analyzer (Bayer). Part of the pancreata from the NOD mice was snap-frozen and stained with anti-insulin and anti-CD45 antibodies (Abs) as published [24]. For cellularity analysis, the islets from the rest of the pancreata were smashed, counted and stained with the antibodies against leukocyte markers, and underwent FACS analysis.

### 2.2. Transwell Migration Assay

JK-EF1*α*-CCR5 cells, a Jurkat clone stably transduced by a lentiviral vector harbouring a CCR5 gene under the control of EF1*α* promoter [35], Jurkat cells (ATCC No. TIB-152), and splenocytes from 7-week-old NOD mice were used to measure CXCR4- and CCR5-mediated chemotaxis as previously described [35]. Briefly, the cells, which were pretreated with vehicle (0.1% DMSO), catenarin, and/or its derivatives for 1 h, were subjected to transwell migration assays with or without chemokine for an additional 4 h. The migrated cells were quantified using hematocytometry. The migration index (MI) was obtained from the formula: MI = 100 × (number of anthraquinone-treated cells migrating toward the chemokine and number of anthraquinone-treated cells migrating toward the medium)/(number of vehicle-treated cells migrating toward the chemokine and number of vehicle-treated cells migrating toward the medium). To evaluate the effect of catenarin on the chemotaxis-mediated by MKK6 and MKK7, Jurkat cells were transiently electroporated with pcDNA3-HA-MKK6EE [36] or pMEV2HA-MKK7EE (Biomyx Technology, CA, USA). Total lysates underwent immunoblot with anti-MKK6, anti-MKK7, and anti-p85 Abs.

### 2.3. *μ*-Slide Migration Assay [37]

The *μ*-slide migration assay was analyzed according to the manufacturer's instructions (IBIDI GmbH, Germany). Briefly, Jurkat cells were loaded in the *μ*-slide, which was precoated with fibronectin. Time-lapse video microscopy was used to record the position of cells at 5-min intervals. Images of cell movement to the chemokine (0.5 *μ*g/mL) or vehicle (0.1% DMSO) were assessed by the ImageJ plugin software for analysis of track data.

### 2.4. Detection of Intracellular Calcium

Ten million JK-EF1*α*-CCR5 cells and Jurkat cells were pretreated with vehicle (0.1% DMSO), catenarin (1 *μ*g/mL), SB202190 (a p38 inhibitor, 3.3 *μ*g/mL), or SP600125 (a JNK inhibitor, 2.2 *μ*g/mL) for 1 h. After washing, the cells were preloaded with Fura 2-AM (5 *μ*M) in modified Krebs-Henseleit buffer (1.3 mM CaCl_2_, 20 mM Hepes, 15 mM glucose, and 2% BSA at pH7.4) for 30 min at 25°C for 1 h. After extensive washing, the cells were stimulated with a dose (100 ng/mL) of MIP-1*β* or SDF-1*β*. Intracellular calcium was measured using a fluorescence spectrophotometer (CAF 110, Jasco, Tokyo, Japan) at the excitation wavelengths of 340 and 380 nm and emission wavelength of 500 nm. The ratio of fluorescence intensity at 340 nm to that at 380 nm represents the level of intracellular calcium.

### 2.5. FACS Analysis

JK-EF1*α*-CCR5 cells and Jurkat cells, which had already been pretreated with vehicle (0.1% DMSO) or catenarin (1 *μ*g/mL) for 1 h, were stained with isotype Ab, FITC-conjugated anti-CCR5 Ab, or anti-CXCR4 Ab plus FITC-conjugated secondary Ab. The cells were subjected to FACS analysis and results were analyzed using FCS Express software. Pancreatic islets were smashed and islet cells were collected. The cells were stained with anti-CCR5 Ab or anti-CXCR4 Ab and underwent FACS analysis.

### 2.6. Immunoblot

JK-EF1*α*-CCR5 cells and Jurkat cells, which had already been pretreated with vehicle (0.1% DMSO) or catenarin (1 *μ*g/mL) for 1 h, were stimulated with MIP-1*β* or SDF-1*β* for 0, 5, 10, and 15 min. Total lysates from the cells were subjected to SDS-PAGE and blotted with Abs against the MAPKs or their phosphorylated forms. Proteins were visualized using ECL kits and detected using ChemiGenius image analysis system (Syngene, Cambridge, UK). The relative intensities of the protein bands were quantitated using Syngene GeneTools software.

### 2.7. Statistical Analysis

Data from three or more independent experiments are presented as mean ± standard error (SE). ANOVA and Mann-Whitney *U* Test were used for statistical analysis between control and treatment groups in *in vitro* and *in vivo* studies, respectively.

### 2.8. Reagents and Cells

Catenarin was purchased from ChromaDex (CA, USA). DMSO, acetylsalicylic acid, emodin, physcion, rhein, resveratrol, and secondary Abs against mouse and rabbit sera were purchased from Sigma (MO, USA). WST-1 assay was purchased from Roche (IN, USA). Human chemokines (SDF-1*β* and MIP-1*β*), mouse chemokines (SDF-1*α* and MIP-1*β*), FITC-conjugated anti-CCR5 Ab, anti-CXCR4 Ab, isotype Ab, and FITC-conjugated secondary Ab were purchased from R&D Systems (Minneapolis, MN, USA), and anti-insulin Ab and anti-p85 were from Santa Cruz (CA, USA). Antibodies against MAPKs, MKK6/7 and their phosphorylated proteins were purchased from Cell Signaling (MA, USA), and anti-CD45, anti-CD4, anti-CD8, anti-CD11c, anti-B220, anti-CD68, and anti-CD49 Abs were from BD Biosciences (CA, USA). PVDF membrane and enhanced chemoluminescence detection reagent were purchased from GE healthcare (Piscataway, NJ, USA), and Fura 2-AM was from Molecular Probe (OR, USA).

## 3. Results

### 3.1. Catenarin Inhibits CCR5- and CXCR4-Mediated Chemotaxis in Jurkat Cells and Splenocytes

**Figure 1:**
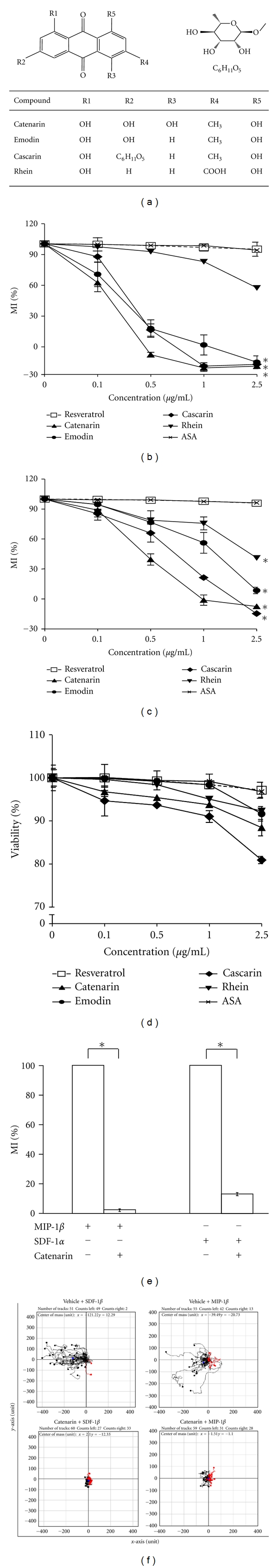
Effect of anthraquinones on chemotaxis and cell viability (a). Chemical structure of 4 anthraquinones: catenarin, emodin, cascarin and rhein (b) and (c). JK-EF1*α*-CCR5 (b) and Jurkat cells (c) were incubated with the indicated doses (0–2.5 *μ*g/mL) of catenarin (▲), emodin (●), cascarin (*◆*), rhein (▾), acetylsalicylic acid (x) or resveratrol (□), a negative control, for 1 h. The cells were subjected to chemotaxis assays in the presence or absence of MIP-1*β* (100 ng/mL) (b) and SDF-1*β* (100 ng/mL) (c) for 4 h. The migration index (MI) is presented as percentage (%). Data from 3 independent experiments are expressed as mean ± SE. *P* (*) < 0.05 (d). JK-EF1*α*-CCR5 cells (10^5^ per well) were pretreated with the indicated doses (0–2.5 *μ*g/mL) of resveratrol, acetylsalicylic acid, or the anthraquinones for 1 h; and cell viability was analyzed by WST-1 assay. Data from 3 independent experiments are presented as means ± SE. E. NOD splenocytes were preincubated with catenarin (1 *μ*g/mL) or vehicle for 1 h. The cells underwent chemotaxis in the presence or absence of MIP-1*α* (100 ng/mL) and SDF-1*β* (100 ng/mL). Migration index (MI) was measured. Data from 3 independent experiments are expressed as mean ± SE. *P* (*) < 0.05. (f). Track plots showing the directional migration of Jurkat cells (left column) and JK-EF1*α*-CCR5 (right column) and, which were pretreated with vehicle (upper row) or catenarin (0.5 *μ*g/mL, lower row), toward SDF-1*β* (50 ng/mL, left column) or MIP-1*β* (50 ng/mL, right column) in *μ*-slide migration assays. Each started in the center of the diagram with SDF-1*β* or MIP-1*β* on left side. Cells were tracked for 8 h. Tracks of cells moving toward and away from the chemokines are highlighted in black and red, respectively.

Leukocyte migration (insulitis) is associated with *β*-cell loss in T1D [6] and mediated by chemokines and their receptors [37]. In this study, we first evaluated the medicinal effect of catenarin and its 3 derivatives (Figure 1(a)) on chemokine-directed chemotaxis in Jurkat cells and their stable clone, JK-EF1*α*-CCR5 cells, using transwell migration assays. CXCR4 and CCR5 chemokines were selected here because both are expressed in naïve and activated T cells, a dominant type of leukocytes in eliciting T1D. The half maximal inhibitory concentration (IC_50_) of catenarin, emodin, cascarin, and rhein on chemotaxis, triggered by CCR5 ligand, MIP-1*β*, in JK-EF1*α*-CCR5 cells was 0.24, 0.36, 0.39, and 3.1 *μ*g/mL, respectively (Figure 1(b)). In contrast, the IC_50_ of catenarin, emodin, cascarin and rhein on chemotaxis, triggered by CXCR4 ligand, SDF-1*β*, in Jurkat cells was 0.46, 1.8, 0.86, and 2.8 *μ*g/mL, respectively (Figure 1(c)). Of note, catenarin, the most potent anthraquinone tested in this study, inhibited CCR5- and CXCR4-implicated chemotaxis in a dose-dependent manner (Figure 1). In contrast, two anti-inflammatory compounds, resveratrol and acetylsalicylic acid, had no significant antichemotactic activity in Jurkat cells (Figures 1(b) and 1(c)). Moreover, the viability of JK-EF1*α*-CCR5 cells and Jurkat cells exposed to the anthraquinones at a dose of 0 to 2.5 *μ*g/mL for 1 h is >80% based on WST-1 assays (Figure 1(d) and data not shown). These data suggest that the suppression of CCR5- and CXCR4-implicated chemotaxis by the anthraquinones is not due to cytotoxicity. We also examined the effect of catenarin on MIP-1*β* and SDF-1*α*-mediated chemotaxis in NOD splenocytes. The data showed that catenarin significantly inhibited CXCR4- and CCR5-mediated chemotaxis in splenocytes (Figure 1(e)) in a similar way as it did in Jurkat cells (Figures 1(b) and 1(c)).

We further used *μ*-slide migration assays to record the directional track of Jurkat cells moving toward chemokines [38]. Track plot data showed that vehicle-treated Jurkat cells and JK-EF1*α*-CCR5 cells migrated towards the left side of the *μ*-slide where SDF-1*β* and MIP-1*β* were loaded, respectively (upper row, Figure 1(f)). Strikingly, catenarin treatment abolished the movement of Jurkat cells and JK-EF1*α*-CCR5 cells toward SDF-1*β* and MIP-1*β* (lower row, Figure 1(f)). We, for the first time, demonstrate that catenarin and its analogs could inhibit CXCR4- and CCR5-implicated cell migration as shown by the two types of migration assays. The data suggest that catenarin effectively inhibits leukocyte chemotaxis.

### 3.2. Catenarin Suppresses Leukocyte Migration, Islet Destruction, and T1D in NOD Mice

**Figure 2:**
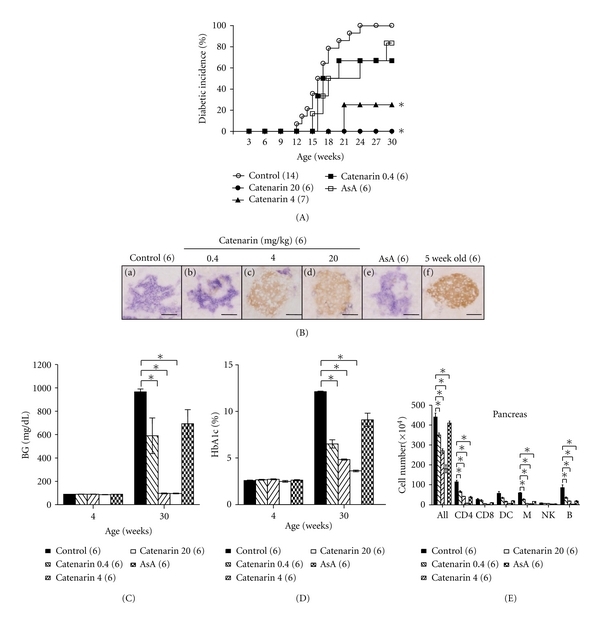
Effect of catenarin on prevention of diabetes in NOD mice. (A) The cumulative diabetic incidence, from 3 to 30 weeks of age, was monitored in female NOD mice treated with vehicle (control), catenarin (0.4, 4, and 20 mg/kg) or acetylsalicylic acid (ASA, 40 mg/kg), 3 times per week, from 4 to 30 weeks. The number of mice per group is indicated in the parentheses. (B) Histological images of the pancreata of NOD mice. The pancreata of 30-week-old NOD mice (a)–(e) and diabetes-free 5-week-old NOD mice (f) were fixed and double-stained with anti-insulin (*β* cells, brown) and anti-CD45 (leukocytes, purple) Abs. Scale bar, 100 *μ*m. (C) and (D) The blood glucose level (C) and Hb_A1C_ percentage (D) of the above-mentioned mice at the age of 4 weeks (before treatment) and 30 weeks were measured. (E), (F), and (G). The number of total cells and leukocyte subsets of pancreatic islets (E) from NOD mice (A) was determined. Anti-CD4 (CD4^+^ T cells), anti-CD8 (CD8^+^ T cells), anti-CD11c (DCs, dendritic cells), anti-B220 (B cells), anti-CD68 (macrophages), and anti-CD49 (NK cells) Abs were used to determine leukocyte subsets.

We turned to investigate the *in vivo* effect of catenarin on T1D, an inflammatory disease caused by the destruction of pancreatic *β* cells by leukocytes, in NOD mice. We first examined the cumulative incidence of diabetes in NOD mice treated with or without catenarin for 30 weeks. All NOD mice (100%) treated with vehicle spontaneously developed T1D from the age of 24 weeks (Figure 2(a)). In contrast, 83% of 30-week-old NOD mice treated with the anti-inflammatory drug, acetylsalicylic acid [25], developed T1D (Figure 2(a)). Of note, catenarin at 0.4, 4, and 20 mg/kg reduced diabetes in the age-matched mice by 33%, 86%, and 100%, respectively (Figure 2(a)). The data showed that catenarin prevented T1D in NOD mice to a greater extent than acetylsalicylic acid, a Cox-2 inhibitor. Next, we checked islet architecture and leukocyte migration in the pancreata of these mice (Figure 2(b)). Severe islet destruction and leukocyte infiltration was found in the pancreata of NOD mice treated with vehicle, 0.4 mg/kg catenarin and acetylsalicylic acid (Figure 2(b)). In marked contrast, a marginal-to-modest islet destruction and leukocyte infiltration were found in the pancreatic islets of NOD mice treated with catenarin at 4 mg/kg and over (Figure 2(b)). These histopathological data supported that catenarin could strongly maintain the islet integrity and suppress the leukocyte migration in 30-week-old NOD mice.

The levels of blood glucose and Hb_A1C_ represent short-term and long-term glycemic control in diabetic mice. We found that 4-week-old NOD mice had a normal level of blood glucose and Hb_A1C_ (Figures 2(c) and 2(d)). However, 30-week-old NOD mice treated with vehicle, 0.4 mg/kg catenarin and acetylsalicylic acid had an elevated level of blood glucose and Hb_A1C_ (Figures 2(c) and 2(d)). In contrast, NOD mice treated with catenarin at 4 mg/kg and over effectively reduced the level of blood glucose and Hb_A1C_ (Figures 2(c) and 2(d)). Our data demonstrate that the maintenance of blood glucose by catenarin is consistent with the islet integrity.

Catenarin suppressed leukocyte infiltration into the pancreas in the late-stage diabetes (30-week-old mice) (Figure 2(b)). Similarly, it reduced leukocyte infiltration and the severity of insulitis in the early-stage diabetes (12-week-old NOD mice) (data not shown). The data suggest that catenarin inhibits the leukocyte migration into pancreas during T1D development.

We further analyzed the number and type of leukocytes in pancreatic islets of 30-week-old NOD mice using FACS analysis. In agreement with the effect of catenarin on leukocyte infiltration as shown in histopathological data (Figure 2(b)), catenarin significantly reduced the number of total leukocytes, CD4^+^ T cells, macrophages, and B cells in the pancreatic islets of NOD mice in a dose-dependent fashion (Figure 2(e)). Catenarin also reduced the number of CD8^+^ T cells and dendritic cells although this decrease was not statistically significant (Figure 2(e)). However, acetylsalicylic acid had no significant decrease in the number of leukocytes and their subsets (Figure 2(e)). The data conclude that catenarin inhibits the leukocyte migration to pancreatic islets of NOD mice (Figure 2) in a similar way as it inhibits the chemotaxis of Jurkat T cells and splenocytes (Figure 1).

Overall, the data demonstrate that catenarin prevents the leukocyte migration into the pancreas (i.e., insulitis) and, consequently, reduces islet destruction and T1D in NOD mice. Besides, catenarin is more effective than acetylsalicylic acid in T1D prevention.

### 3.3. Catenarin Does Not Affect Surface Expression of CCR5 and CXCR4 Receptors

**Figure 3:**
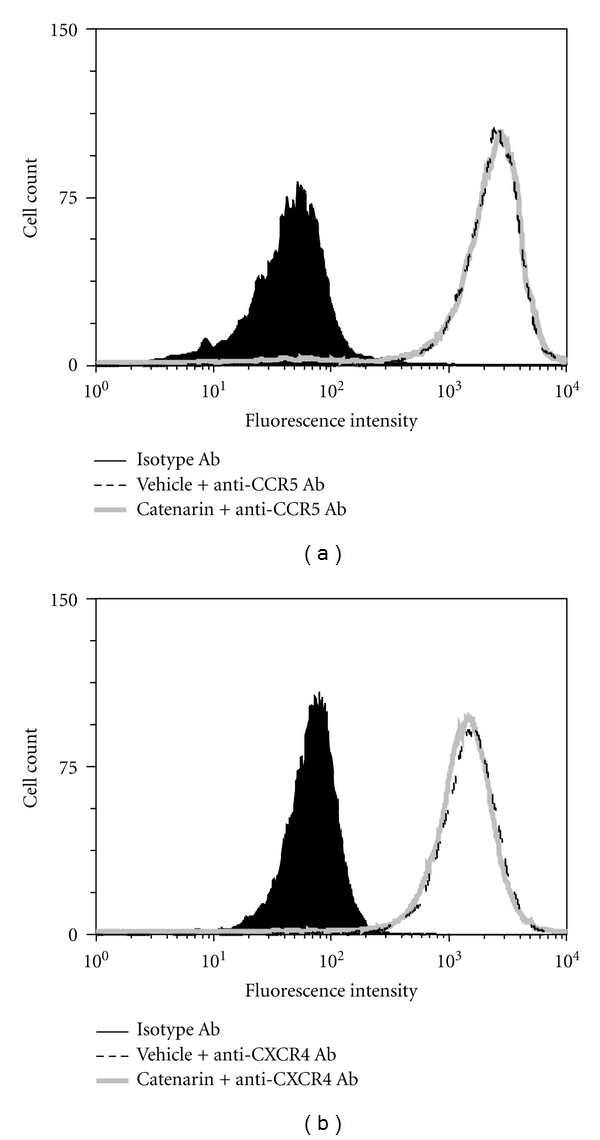
Effect of catenarin on expression level of CCR5 or CXCR4 (a). JK-EF1*α*-CCR5 cells were incubated with vehicle or catenarin (1 *μ*g/mL) for 1 h. The cells were stained with FITC-conjugated anti-CCR5 Ab or isotype Ab plus FITC-conjugated secondary Ab. Expression level of CCR5 was indicated by fluorescence intensity. The data are representative of 4 independent experiments. (b) The same procedure was followed as in (a) except that Jurkat cells and anti-CXCR4 Ab were used.

Reduction of chemotaxis by catenarin may be due to a decrease in cell surface expression of CCR5 and CXCR4 receptors due to the internalization of these receptors induced by catenarin. To test this possibility, catenarin was used to treat JK-EF1*α*-CCR5 cells or Jurkat cells. After treatment for 1 h, the surface expression level of CCR5 on JK-EF1*α*-CCR5 cells and CXCR4 on Jurkat cells was measured using FACS analysis. No difference was observed in the expression of CCR5 and CXCR4 on the cell surface (Figure 3), thus ruling out the possibility that catenarin works at the level of the chemokine receptors.

### 3.4. Catenarin Inhibits Calcium Mobilization in CCR5 and CXCR4 Pathways

**Figure 4:**
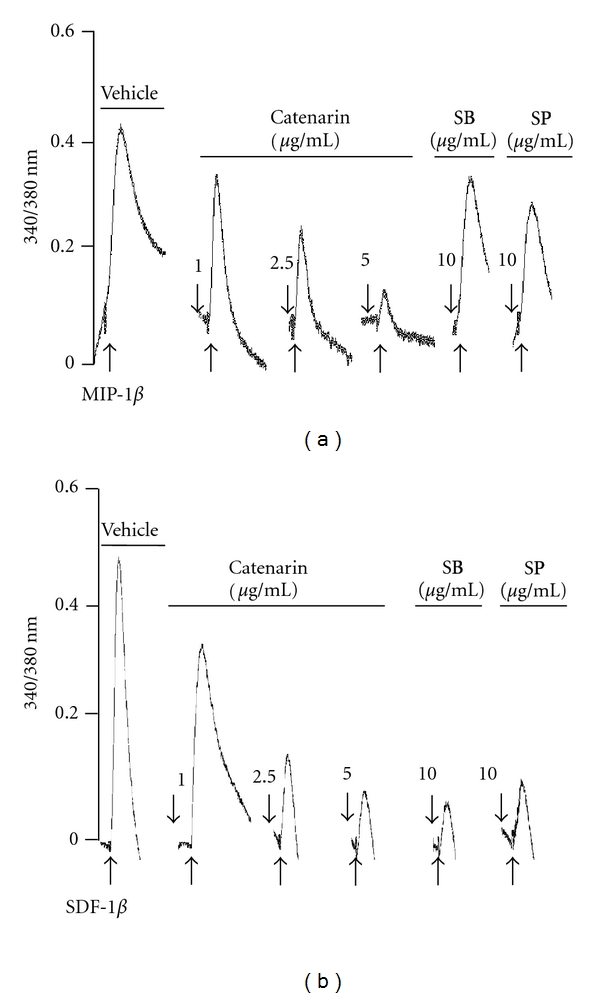
Effect of catenarin on calcium mobilization elicited by MIP-1*β* or SDF-1*β*. Ten million JK-EF1*α*-CCR5 cells (a) and Jurkat cells (b) were pretreated with vehicle, catenarin (1–5 *μ*g/mL), SB202190 (SB), or SP600125 (SP) for 1 h. After Fura 2-AM loading, the cells were stimulated with MIP-1*β* (a) or SDF-1*β* (b). Level of intracellular calcium, as shown by the 340/380 nm ratio, was detected using a fluorescence spectrophotometer.

Calcium is known to be a secondary messenger downstream of some chemokine receptors in T cells [21]. Thus, we examined the effect of catenarin, SB202190 (a p38 inhibitor) and SP600125 (a JNK inhibitor) on calcium mobilization triggered by MIP-1*β* or SDF-1*β*. Interestingly, catenarin dramatically reduced calcium influx triggered by both chemokines in a dose-dependent manner (Figure 4). The p38 inhibitor and JNK inhibitor significantly diminished MIP-1*β*- and SDF-1*β*-elicited calcium influx in JK-EF1*α*-CCR5 and Jurkat cells, respectively (Figure 4). The above data imply that the inhibition of CCR5- or CXCR4-mediated chemotaxis by catenarin involves calcium and the MAPKs, p38, and JNK.

### 3.5. Catenarin Inhibits the Activation of MAPK Cascades in CCR5 and CXCR4 Pathways

**Figure 5:**
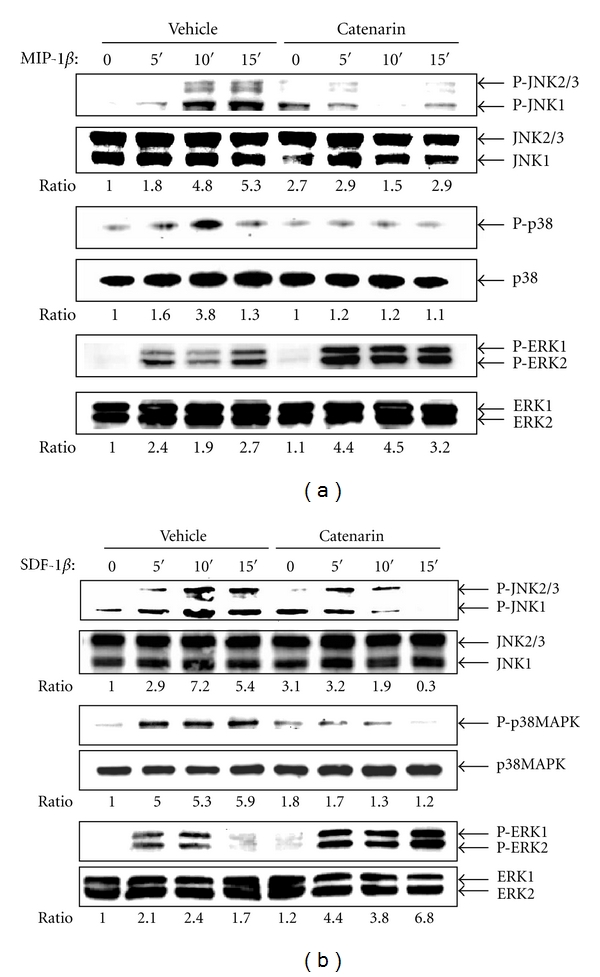
Effect of catenarin on MAPK phosphorylation elicited by MIP-1*β* or SDF-1*β*. One million JK-EF1*α*-CCR5 cells (a) and Jurkat cells (b) were pretreated with vehicle or catenarin (1 *μ*g/mL) for 0, 5, 10, and 15 min, followed by the addition of (a) MIP-1*β* (100 ng/mL) or (b) SDF-1*β* (100 ng/mL). Total lysates were immunoblotted with the Abs against MAPKs (p38, JNK, and ERK) or their phosphorylated proteins. Representative data and the ratio of the signal of the phosphorylated MAPKs to that of total MAPKs are shown.

MAPKs are known to function the downstream of chemokine receptors [21]. Therefore, we checked the effect of catenarin on MAPK family, p38, JNK, and ERK1/2, in the CCR5 and CXCR4 pathways. We found that catenarin at 1 *μ*g/mL inhibited the phosphorylation of the p38 and JNK in JK-EF1*α*-CCR5 and Jurkat cells in response to MIP-1*β* (Figure 5(a)) and SDF-1*β* (Figure 5(b)). However, catenarin slightly increased in the phosphorylation of ERK1/2 (Figure 5). The data suggest that the antichemotactic effect of catenarin is consistent with the inhibition of p38 and JNK by catenarin, but not the activation of ERK1/2 by catenarin.

**Figure 6:**
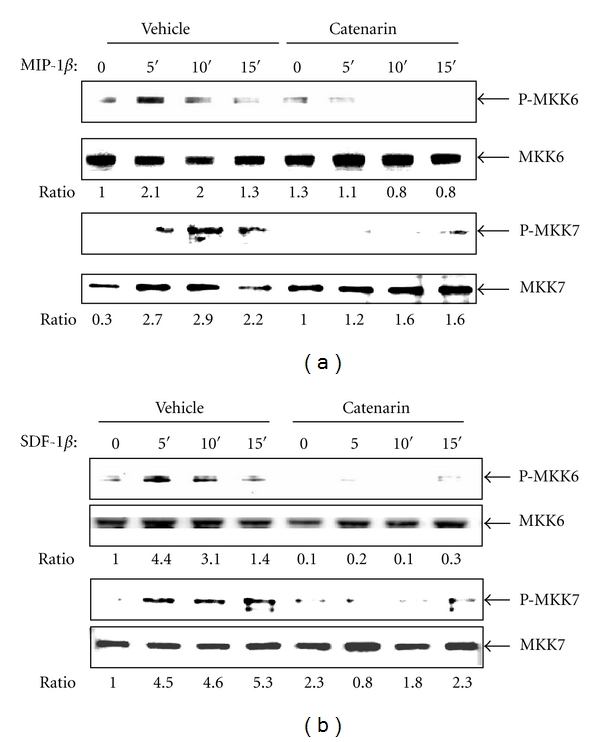
Effect of catenarin on MKK6 and MKK7 phosphorylation elicited by MIP-1*β* or SDF-1*β*. JK-EF1*α*-CCR5 cells (a) and Jurkat cells (b) received the same treatment as described in Figure 5. Abs against MKK6, MKK7, and their phosphorylated proteins were used in immunoblot.

MAPKs can be activated by their upstream regulators, the MAPK kinases (MKKs). MKK3 and MKK6 regulate the activation of p38, whilst MKK4 and MKK7 regulate the activation of JNK [39, 40]. We further examined the effect of catenarin on the activation of MKK6 and MKK7. Catenarin significantly reduced the phosphorylation of MKK6 in K-EF1*α*-CCR5 cells triggered by MIP-1*β* and Jurkat cells triggered by SDF-1*β* (upper panels, Figures 6(a) and 6(b)). Similarly, catenarin significantly decreased the phosphorylation of MKK7 by either chemokines (lower panels, Figures 6(a) and 6(b)). These data confirm that catenarin inhibits MAPKs, p38 and JNK, and their upstream regulators, MKK6 and MKK7.

**Figure 7:**
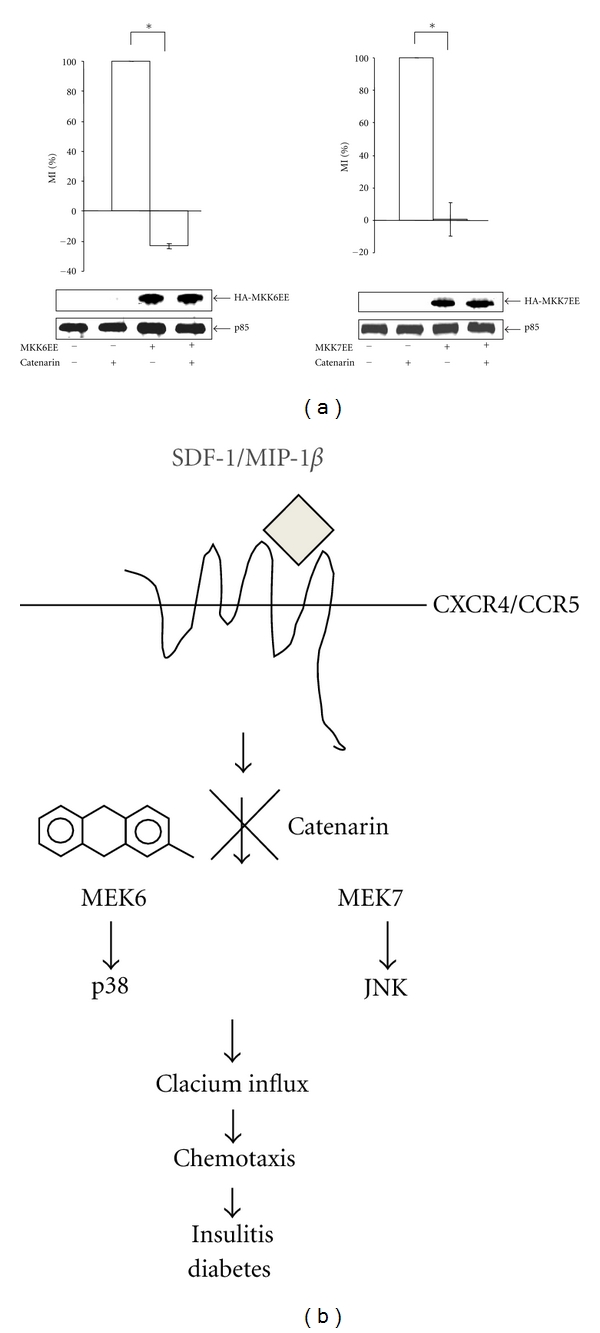
Effect of catenarin on MKK6- or MKK7-mediated migration (a). Jurkat cells were electroporated with pcDNA3-HA-MKK6EE or pMEV2HA-MKK7EE. The cells underwent chemotaxis assay, and the migration index (MI) was determined (upper panel). Data from 3 independent experiments are expressed as mean ± SE.* P* (*) < 0.05. The expression level of MKK6EE, MKK7EE, and p85, an internal control, was determined by immunoblot using anti-MKK6, anti-MKK7, and anti-p85 Abs (lower panel) (b). Scheme describing the molecular mechanism by which catenarin inhibits leukocyte migration and, thus insulitis in NOD mice. Catenarin inhibits leukocyte migration mediated by CCR5 and CXCR4 via the inactivation of MAPKs (p38 and JNK), MKKs (MKK6 and MKK7), and calcium mobilization.

To further prove that catenarin inhibited chemotaxis via the signaling cascade of MKKs and MAPKs, we tested the effect of catenarin in Jurkat cells, which were transfected with an active mutant of MKK6 or MKK7. Accordingly, catenarin significantly abolished Jurkat cell migration mediated by MKK6 and MKK7 (Figure 7(a)). Similar results were observed in primary CD4^+^ T cells with the overexpression of MKK6 or MKK7 (data not shown). Overall, these data suggest that catenarin inhibits CCR5- and CXCR4-implicated chemotaxis via the inactivation of the MKK/MAPK pathway and calcium influx (Figure 7(b)).

## 4. Discussion and Conclusions

In this study, we investigated the anti-inflammatory effect and mechanism of catenarin and/or its analogs using human Jurkat cells, mouse splenocytes, and NOD mice. We demonstrate that catenarin, the most potent anthraquinone tested in our study, strongly suppresses leukocyte migration in the diabetes. Moreover, mechanistic studies showed that catenarin, as well as probably its analogs, acts by targeting the MKK/MAPK signaling cascades and calcium downstream of CCR5 and CXCR4 receptors, expressed in leukocytes.

Naturally occurring anthraquinone compounds are claimed to have a variety of bioactivities. A majority of the literature demonstrate the action of the anthraquinones for constipation [41–43] and cancers [3, 28, 44]. However, their role in inflammation is poorly studied. Here, CXCR4- and CCR5-implicated chemotaxis in Jurkat T cells was employed as an *in vitro* system to investigate the anti-inflammatory effect and action of the anthraquinones. With this system, we showed the antichemotactic effect (Figure 1) and mechanism of catenarin and its anthraquinone analogs in T cells (Figures 3–7). In sharp contrast, two anti-inflammatory compounds, resveratrol [45] and acetylsalicylic acid [25] could not inhibit chemotaxis (Figures 1 and 2). Clearly, not all anti-inflammatory compounds can effectively suppress leukocyte chemotaxis, suggesting the unique anti-chemotactic feature of catenarin and its analogs in leukocytes (Figure 1). Inhibition of chemotaxis in leukocytes by catenarin was further confirmed by the reduction of insulitis and diabetes in NOD mice (Figure 2 and data not shown). The anti-inflammatory action of catenarin in this study is consistent with the findings showing that emodin, the most extensively studied anthraquinone, inhibited hepatitis [30], pancreatitis [32], and NF-*κ*B [33] in different model systems. In addition, diacerhein, a drug used for osteoarthritis that has an anthraquinone structure [34], showed beneficial effects on T1D in NOD mice [46]. The data suggest that catenarin and its anthraquinone analogs are prophylactically useful for T1D and, probably, other inflammatory disorders. Of note, catenarin has higher antichemotactic activity than emodin, cascarin, and rhein. Interestingly, this activity seems to be related to the number of hydroxyl group at R2 and R3 in anthraquinones (Figure 1).

Chemokines and their receptors are potential drug targets in inflammatory diseases such as T1D [14, 47]. CXCR4 is broadly expressed in all the leukocytes and CXCR5 is expressed in macrophages and activated T cells, a pivotal player in autoimmune destruction of pancreatic *β* cells [17]. Both receptors are known to regulate T1D, although their protective roles in T1D are controversial [7, 18, 19, 48]. In view of the importance of CCR5 and CXCR4 in T1D, in this study we explored the anti-inflammatory effect of catenarin based on CCR5- and CXCR4-implicated migration assays. Surprisingly, catenarin and its analogs dose-dependently inhibited CCR5- and CXCR4-mediated chemotaxis in JK-EF1*α*-CCR5 and Jurkat cells, respectively (Figure 1). This inhibition in human Jurkat T cells was further confirmed in mouse splenocytes and NOD mice (Figures 1 and 2). The data also suggest that the antichemotactic effect of catenarin and its analogs is not limited to T cells and exists in other leukocytes.

All chemokine receptors belong to the G-protein-coupled receptor family. Upon chemokine binding, the receptor induces G*α* activation and leads to a signaling cascade that comprises activation of tyrosine kinases, serine/threonine kinases, and phospholipases, and an increase in secondary messengers (calcium, diacylglycerol, and phosphatidylinositol) [21, 49]. In this study, catenarin failed to affect the surface expression level of CCR5 and CXCR4, suggesting that catenarin does not target the receptors themselves (Figure 3). However, catenarin suppressed the activation of chemokine-receptor downstream mediators, MKK6/p38 and MKK7/JNK, and calcium mobilization (Figures 4, 5, and 6). These data suggest that catenarin targets the signaling molecules downstream of CCR5 or CXCR4. Further, the suppression of MKK6- and MKK7-mediated chemotaxis by catenarin suggests the possibility that catenarin targets the signaling molecule(s) upstream of MKKs (Figure 7). The data shown in Figures 5 and 6 suggest that catenarin acts as a kinase inhibitor. Our data revealed that catenarin specifically inhibited the activation of MKK6/p38 and MKK7/JNK in CXCR4 and CCR5 pathways. Controversially, catenarin slightly increased the activation of ERK1/2 in Jurkat cells (Figure 5). The findings partially correlate with the literature indicating that emodin, an analog of catenarin, inhibited the phosphorylation of VEGFR-2 and its downstream effector molecules such as p38, ERK1/2, FAK, and Akt in endothelial cell line [50, 51]. The discrepancy between the activation of ERK1/2 by catenarin and the inhibition of ERK1/2 by emodin is due to the difference of anthraquinone structure because emodin did inhibit CXCR4-mediated ERK1/2 activation in Jurkat cells (data not shown). Most of the literature suggests that calcium mobilization is located upstream of the activation of MAPKs [52]; however, some studies have suggested that MAPKs control intracellular calcium concentration [53]. We found that P38 and JNK inhibitors suppressed calcium mobilization in CXCR4 and CCR5 pathways (Figure 4), which argues for the possibility that MAPKs control calcium mobilization (Figure 7(b)).

In conclusion, we, for the first time, showed that catenarin inhibited CXCR4- and CCR5-implicated leukocyte migration, islet destruction, and diabetes in NOD mice. This inhibition involved the inactivation of the MKK6/p38 and MKK7/JNK signaling cascades and the reduction of calcium influx in the CCR5 and CXCR4 pathways in T cells. This proof-of-concept study demonstrates the effectiveness of catenarin against T1D and molecular mechanism of catenarin in T cells and, probably, other leukocytes.

## Abbreviations

Ab: Antibody
Cox-2: Cyclooxygenase-2
DMSO: Dimethyl sulfoxide
GPCR: G-protein-coupled receptor
Hb_A1C_: Glycosylated hemoglobulin A1C
MAPK: Mitogen-activated protein kinase
MIP-1*β*: Macrophage inflammatory protein-1*β*
MKK: MAPK kinase
NOD: Nonobese diabetic
SDF-1*β*: Stromal cell-derived factor-1*β*
T1D: Type 1 diabetes.
